# The Journey of Cultures Taken During Revision Joint Arthroplasty: Preanalytical Phase

**DOI:** 10.7150/jbji.32975

**Published:** 2019-05-21

**Authors:** Kier M. Blevins, Karan Goswami, Javad Parvizi

**Affiliations:** Rothman Institute at Thomas Jefferson University, Philadelphia, PA 19017

**Keywords:** culture processing, timing, revision arthroplasty, hip, knee

## Abstract

*Background:* Microbiological culture has been considered the standard for pathogen identification for decades. However, culture is a laborious, time consuming, imperfect and outdated process. This study aims to inform the orthopedic community of the steps and timing of routine culture processing.

*Methods:* We prospectively tracked 103 cultures from 33 revision hip and knee arthroplasty patients between September 2017-February 2018. Times were recorded at intraoperative collection; time of pick up from OR, transportation time; arrival at the laboratory; culture processing and plating time; and time to final result reporting.

*Results:* Of the 103 cultures, 45.6% were processed and incubated in less than two hours, and 54.4% greater than or equal to two hours. The mean time spent in the OR, during transport, and within the laboratory prior to incubation was 0:53, 0:06 and 1:12. The range of time that samples remained at each stage varied considerably in the OR (0:03-3:33), in transit(0:04-0:16), and in the lab prior to incubation(0:26-3:01). The proportion of the total time to incubation attributed to idle time samples spent in the OR after initial sampling was 40.0%. In contrast, transport to the laboratory represented 5.1% of the total time. Idle time in the laboratory represented the greatest share at 54.9%.

*Conclusion:* There is significant variability in the time to transport, process and incubate culture samples. Almost half of the specimens were processed outside the 2-hour recommended window. Surgeons should be aware of idle time during processing and seek to optimize their institutional pathways to maximize culture yield.

## Introduction

Culture remains as the mainstay for pathogen identification 1,2. This process has not changed a great deal since its description by Robert Koch with the first use of serum-based solid media in the 1880's 3,4. It is a labor intensive, technician-dependent process that requires a concise methodology given the correct indications for sampling in order to obtain accurate results 5-8. Even despite meticulous efforts, the rates of suspected culture-negative periprosthetic joint infection (PJI) are as high as 6.4% to 42.1% which can vary greatly depending on the clinical picture 9,10. Furthermore, this process, although imperfect remains the primary means of pathogen detection and is essential for targeted antimicrobial therapy for patients.

A number of factors exert influence upon the culture yield, namely isolation of an infective organism. One of these factors is the time from collection to incubation. The Infectious Disease Society of America (IDSA) recommends that in order to maintain microbial viability and to limit desiccation or death of pathogens, culture samples should be processed promptly within a two-hour window following their collection 11,12. Other key aspects include the quality of the collected specimen, the medium and conditions used for incubation, plating techniques used by laboratory technicians that needs to minimize contamination, incubation period, and numerous other factors.

Research has sought to improve standard culture methods for revision surgery by adding additional steps such as sonication of implants and transfer of joint fluid in blood culture bottles which are known to increase the diagnostic sensitivity of culture for PJI 8,13. Additionally, methods to reduce contamination and circumvent the manual labor involved for determining growth have been implemented with methods utilizing blood culture bottles and automated growth detection. Current studies have shown that the use of blood culture bottles for joint fluid, besides increasing sensitivity, helped decrease the personnel demand and cost for microbiologic culture 8,14. A number of studies have also focused on providing the optimal duration for incubation with regards to cultivating organisms responsible for PJI without providing much attention to the processing time which may be more critical 15-17.

The purpose of this prospective study was to examine the steps that are involved in collection, processing and reporting of the final results of culture samples taken during joint arthroplasty. In particular we were interested to determine what percentages of samples were processed outside the recommended two-hour window from collection to plating.

## Materials and Methods

### Tissue and Fluid Culture Demographics

Following institutional review board approval, culture specimens from patients undergoing revision total hip and knee arthroplasty from September 2017 to February 2018 were prospectively tracked. All samples were obtained at a single academic center and processed at an on-site in-house microbiological laboratory. In order to obtain a representative sample of typical processing times, we tracked samples obtained from revision cases operated on by six board-certified arthroplasty surgeons. Furthermore, a range of different tissue types were tracked for any given revision case including: synovial fluid, synovium, capsular tissue, femoral tissue, tibial tissue, and acetabular tissue.

### Data Collection and Timing

Two stages of times were collected by a single research fellow during different phases of specimen collection and culture. The numbers of tissue handlers from the time of collection to incubation during the first stage were recorded. The first stage was from the specimen's initial collection intraoperatively to placement inside an incubator. The original collection time by the orthopaedic surgeon during the revision case was recorded as the initial start time. The time of operating room pick-up, laboratory arrival (post-transport), processing completion and incubation were recorded. The first stage of culture processing was complete at the time of incubation.

The second stage was for all post-incubator procedures and processing times for manual specimen examination following initial incubation after 18-24 hours for the first day. Times were collected when cultures were manually opened and observed for both mycology and bacterial cultures. The cultures were examined manually for the first week each day and then once more at two weeks for the final result for both aerobic and anaerobic cultures. The mycology cultures were examined twice during the first week of initial culture and once every week following for a total of 4 weeks when the final result was determined.

Descriptive statistics were performed to determine the mean time points and distinctions amongst them. Statistical calculations were determined using IBM SPSS Statistics for Windows, Version 22.0 (Armonk, NY).

## Results

Of the 103 cultures, 45.63% (*n*=47) were processed and incubated in a time frame of less than two hours. The remaining 54.36% (*n*=56) were incubated at a time greater than or equal to two hours from the time of initial specimen collection in the OR. Of the 103 samples collected from 33 patients, 44 samples for culture were from TKA revision surgeries and 59 were from THA revision surgeries. The mean number of culture samples per patient was 3.22 (*SD*=0.98; Range 2 to 6).

The average OR idle time for the samples was 59.1 minutes (*SD=*42.4) for THA revision surgery and 46.5 minutes (*SD*=24.9) for TKA revision surgery (p=0.082).

The average time spent in the OR, transport, and the laboratory before incubation was 0:53 (*SD*=0:36), 0:06 (*SD*=0:02) and 1:12 (*SD*=0:44), respectively. These times varied largely with the minimum and maximum time in the OR being 0:03 and 3:37, in transport being 0:04 and 0:16, and in the lab prior to incubation being 0:26 and 3:01, respectively. The total time to incubation for samples ranged widely from 0:46 to 4:33. The mean difference in time to incubation for specimens taken from the same patient was 0:16 (*SD*=0:21). The results for each pre-incubation time phase are displayed in Table 1.

**Figure 1:**
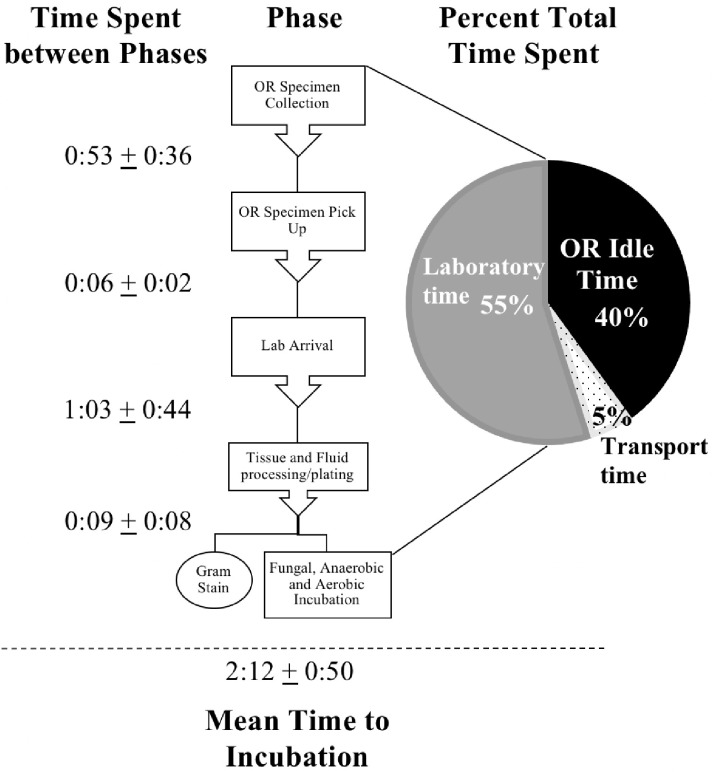
Percent Breakdown of Time by Phase Before Incubation

The average number of individuals who handled specimen from the time of collection up until incubation was 5.99 workers (*SD*=0.47). The mean time to incubation after a specimen was processed and plated onto their specified medium was 9 minutes (*SD*=8). Samples for mycology were always ground while specimens for bacterial cultures were only cut and partially homogenized before processing. For the entire pre-incubation process, the mean percentage of time spent in the OR after initial collection was 40.0%, for transport was 5.1%, and in the laboratory prior to incubation was 54.9% of the total mean time to incubation. These percentages by phase are displayed in Figure 1.

## Discussion

Identification of a pathogen and determining its susceptibility to antimicrobials are critical steps in the management of patients with PJI. Traditional microbiology techniques are still widely employed to isolate the infective organism(s). The latter process is reliant on timely arrival of the culture samples in the laboratory, and prompt plating and processing of the specimen. Since the first description of culture methods in 1880's by Koch, not much has changed in this field with regard to principles. Even with substantial technological advancements and innovations, the process is prone to error in numerous steps that includes variability in sample collection, time from collection to processing, the techniques of sample processing, the choice and duration of incubation 18-20. Being cognizant of the issues related to culture, the IDSA has published recommendations about processing of culture samples that aims to improve the sensitivity of diagnostic procedures. One of the recommendations of the IDSA is that any samples collected for culture should be processed within two hours of collection in order to minimize desiccation of tissues and improve microbial viability 11,12.

This study demonstrated that almost one-half of the samples were processed outside of the two-hour window. The latter was despite the fact that microbiology laboratories are located within the same building as the operating rooms and our microbiology technicians are trained and required to process samples from orthopedic procedures expeditiously. Thus, the data from our institution most likely represents the best-case scenario. The study also found that there was a wide variability in processing of these samples with some collected specimen sitting in the laboratory for over three hours before being processed.

There are many reasons for the wide variability that was observed in this study. Like any other major academic medical centers, the personnel in the laboratory were at times under heavy work burden that delayed the processing of arrived samples. Our institution has a hierarchy in place for the processing of specimens received in order to adhere to this high requirement and IDSA guidelines for the procurement of specimens for culture and blood culture. Cerebrospinal fluid, ocular specimens, joint fluid and intraoperative specimens are processed before all other samples at the time of arrival. For the revision arthroplasty cases tracked in this study, the intraoperative specimens were given higher priority.

The wide variability that was seen in just about every step of the process, perhaps provides partial explanation to why in almost one-third of proven PJI cases at our institution, an infective organism cannot be isolated. In the IDSA recommendations from 2018, Miller et al. highlight the importance of a 2-hour maximum window for sample processing 11,12. The delay in processing of samples is known to influence microbial viability including viability of common pathogens such as *Staphylococcus aureus* 11. The issue is even more critical for anaerobic and slow growing organisms like *Cutibacterium acnes*. A study by Venturelli et al. assessed pathogen recovery from cultures processed outside and within a 2-hour window 21. This study validated the IDSA's 2-hour guideline by showing that positive culture rates were reduced significantly by 16% when the preanalytical phase extended beyond 2 hours and that overall detection rates were reduced with prolonged times to initial incubation 21. Other literature supporting this stance is from studies with blood cultures and urinary specimen processing that demonstrated altered yield with prolonged transport times greater than 2 -4 hours 22-24. Sautter et al. also showed significant diminution in pathogen recovery when blood culture bottles were held for >24 hours at 4°C or room temperature and for >12 h at 37°C 25.

In our study we noted that the specimens could sit idle in the OR after collection and upon arrival in the lab for upwards of three hours (Table 1). In extreme cases, it took up to four and half hours until an intraoperative sample was completely processed and incubated. Our study highlights that these IDSA guidelines are difficult to achieve even at a major academic institution which is a tertiary referral center for PJI, even despite in-hospital processing of samples within the same institutional building. Furthermore, this raises concerns about the validity and precision of culture reporting from external commercial laboratories that require samples to be transported at great length and outsourced with much lengthier periods of time between collection and processing of specimens for culture.

A potential remedy for the delay during this preanalytical phase may be through the introduction of total laboratory automation (TLA) systems into clinical microbiology labs. Current systems such as Copan's WASPLab and Becton Dickinson's Kiestra TLA are revolutionizing laboratory efficiency through automatizing specimen processing, via standardized robotic plating and streaking methods and digital microbiology 26. Choi et al. demonstrated in their comparison of manual and automated streaking systems, that automated systems reduced laboratory technician handling time without compromising quality of specimens 27.

There are several limitations to our study that we feel should be acknowledged. We did not examine the impact of time on culture yield as this was not the intention of this present study. Secondly, as this was a prospective observational study, the Hawthorne effect may have played a role in the data collection process. The timing data of the study may have been slightly skewed as the laboratory technologists, operating room circulators and specimen transporters were aware of the ongoing study and the tracking of the specimens 28. Third, our sample size was relatively small and the generalizability of our findings may also be brought into question since all specimens were processed in house at a single academic institution from revision joint arthroplasty cases only. Lastly, all timings were recorded by a single observer, with an absence of intra- or inter-rater assessment.

Despite the aforementioned limitations, we feel that the findings of this study shed considerable light on this often-overlooked factor which may influence culture yield, and it provides a realistic representation of typical sample pathway performance to inform the orthopedic community. This is the first study to describe and examine the typical pathway of transport and processing of intraoperative orthopedic specimens, from the time of sampling to incubation. It provides valuable insight into the importance of idle time, and where this is often encountered along a representative pathway of a culture sample. Methods of microbial identification remain a highly time-consuming, labor-intensive, and user-dependent process that is prone to error in various steps. Perhaps it is because of the limitations of culture, especially in orthopedics, that has prompted the American Association of Microbiologists to state that promising molecular techniques, such as next generation sequencing, are likely to replace culture by providing a single all-encompassing method for streamlined and improved micro-organism identification 29. Until these technologies reach widespread clinical usage, the issues with culture need to be recognized and addressed as much as possible.

## Figures and Tables

**Figure 2:**
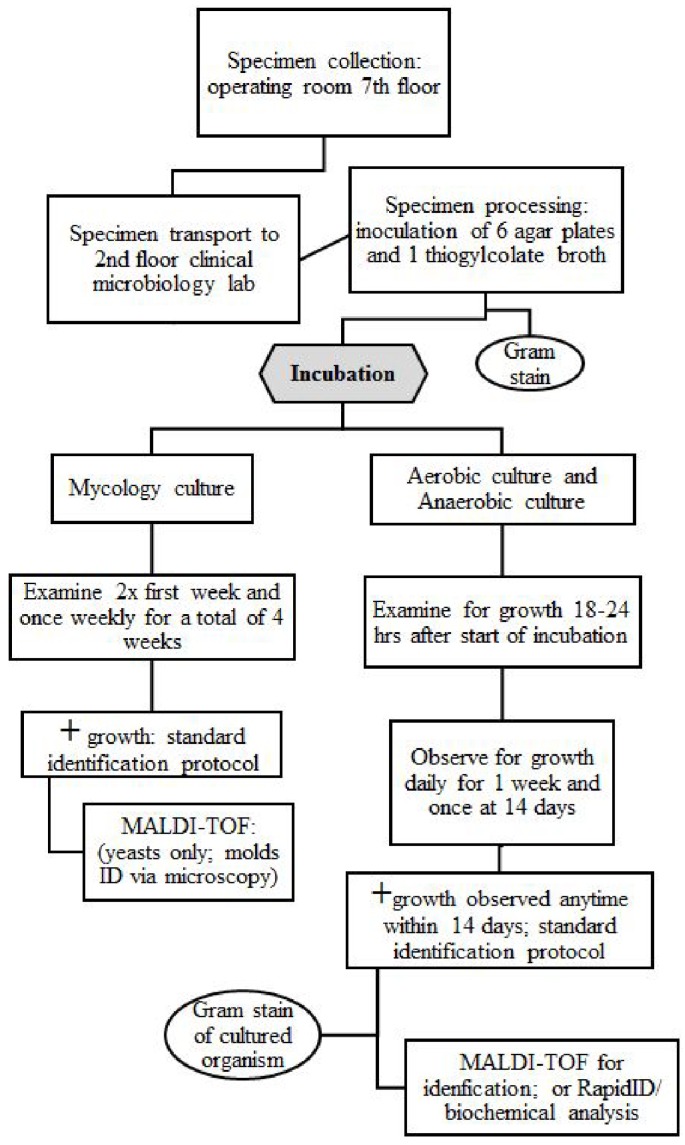
Work Flow Diagram for a Culture

**Figure 3:**
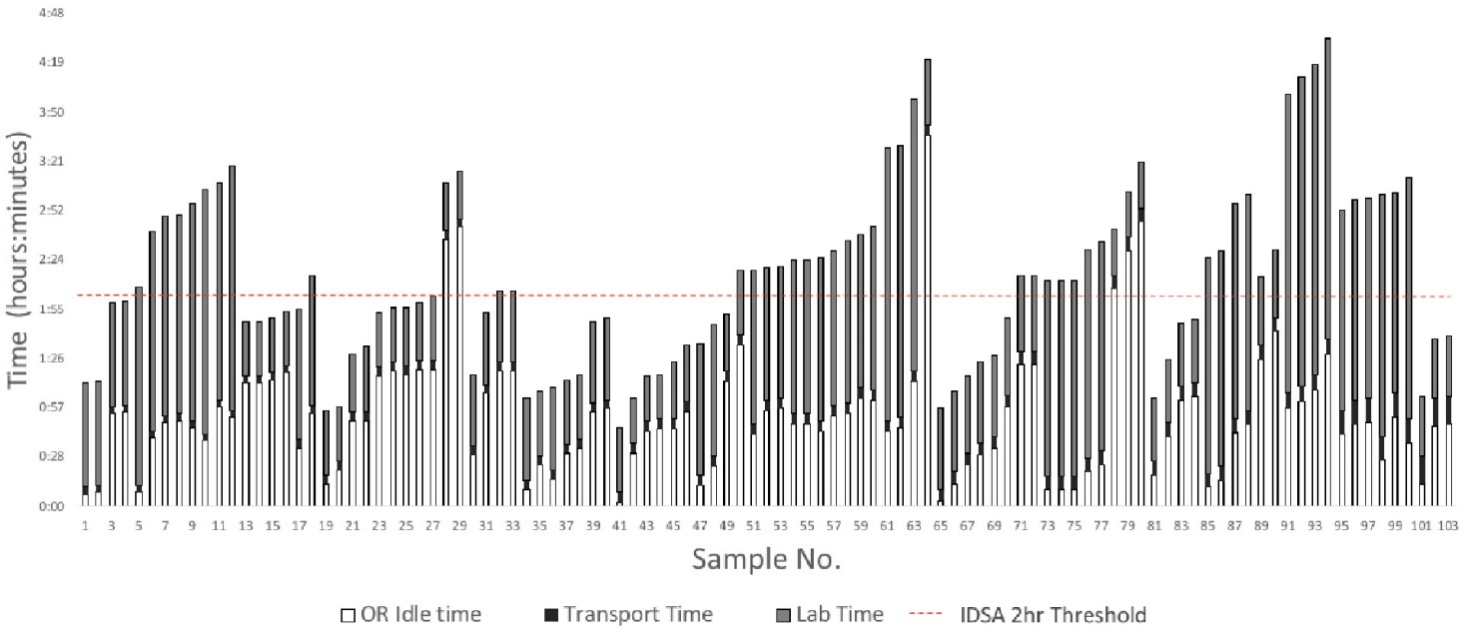
Comparison of Time Spent in Phases Before Incubation

**Table 1:** Results Comparing Time Spent in Phases Before Incubation (n=103)

Phases of Time	Average (hr:min ± SD)	Minimum (hr:min)	Maximum (hr:min)
Operating Room Idle Time	0:53± 0:36	0:02	3.37
Transport Time	0:06± 0:02	0:04	0:16
Laboratory Time	1:12± 0:44	0:26	3:01
Total Time to Incubator	2:12± 0:50	0:46	4:33

**Table 2:** IDSA Culture Recommendations for Prosthetic Joint and Surgical Site infections

Diagnostic procedures	Optimum Specimens	Transport method; Optimal Time to Incubation
Aerobic bacterial culture	minimum 3 intra-operative tissue sample (*Synovial fluid or submit prosthesis in sterile container for sonication protocol)	Sterile container, RT, 2 hours
Anaerobic bacterial culture (incubate cultures up to 14 d; i.e. Cutibacterium species)	Consider submitting infected prosthesis for sonication (minimum 3 intra-operative tissue sample)	Sterile anaerobic transport container, RT, Immediately
Fungal culture	Tissue/biopsy/aspirate	Aerobic transport device Sterile container RT, <2 h

Adapted from A guide to utilization of the microbiology laboratory for diagnosis of infectious diseases: 2013 recommendations by the Infectious Diseases Society of America (IDSA) and the American Society for Microbiology. Common etiologic agents: Streptococcus spp (group A, B and other β hemolytic types), Staphylococcus aureus, Enterobacteriaceae, Psuedomonas Aeruginosa, Corynebacterium, Cutibacterium acnes, Candida spp.*Enterococcus spp which gram stain is not useful. RT= room temp, D = days.

## Abbreviations

PJI: periprosthetic joint infection
IDSA: Infectious Disease Society of America
THA: Total hip arthroplasty
TKA: Total knee arthroplasty
TLA: Total laboratory automation
